# Authors' reply

**DOI:** 10.1590/S1806-37132014000500018

**Published:** 2014

**Authors:** Leonardo de Assis, Mauro César Isoldi

**Affiliations:** Institute of Biosciences, University of São Paulo, São Paulo, Brazil; Federal University of Ouro Preto, Ouro Preto, Brazil

Dear Dr. Eduardo Algranti, 

We would like to thank Dr. Eduardo Algranti for his interest in our paper and for his comments, which greatly contribute to the study in question and to a broader discussion of malignant mesothelioma (MM) in Brazil. 

First, we would like to reply to Dr. Algranti's comments on simian virus 40 (SV40). In the abstract of our article,^(^ 1 ^)^ we state that "the development of MM is strongly correlated with exposure to asbestos and erionite, as well as to simian virus 40". We agree with Dr. Algranti that the statement is incorrect as it is. 

Before our article was edited by the Brazilian Journal of Pulmonology, the statement read "the development of this cancer is strongly correlated with exposure to asbestos and other factors, such as erionite and SV40". In the original sentence (i.e., before the editing of the article), "strongly correlated" referred to "exposure to asbestos" rather than "exposure to asbestos, erionite, and SV40"; however, we recognize that the sentence was ambiguous and open to interpretation. The original sentence might have been clearer if we had placed a comma between the words "asbestos" and "erionite", in an attempt to indicate that "strongly correlated" referred to "asbestos" only; however, we recognize that the sentence would have remained ambiguous and open to interpretation. 

Several studies have strongly correlated asbestos with the development of MM. However, we agree with Dr. Algranti that it is incorrect to state that there is a strong correlation between SV40 and MM. In a recent study conducted by our research group,^(^ 2 ^)^ the role of SV40 in MM was described as follows: 

"Taken altogether, it is still not clear the direct carcinogenic effects of SV40 in MM in humans; however, it is widely accepted the role of SV40 as a co-carcinogenic player in association with asbestos in the development of MM." 

Although the original sentence was no longer ambiguous after the editing of the article, the resulting statement was incorrect. It is indeed incorrect to state that the carcinogenic potential of asbestos and erionite is the same as that of SV40. Therefore, we will request that the Brazilian Journal of Pulmonology correct the sentence in the abstract to read "the development of MM is correlated with exposure to asbestos and other factors, such as erionite and simian virus 40". 

Second, we would like to reply to Dr. Algranti's comments on our description of the role of chrysotile asbestos in the development of MM. We agree with Dr. Algranti's statement that the carcinogenic potential of chrysotile asbestos is lower than that of amphibole asbestos. In fact, in our article,^(^ 1 ^)^ we stated that all types of asbestos are classified as carcinogenic to humans by the International Agency for Research on Cancer (IARC).^(^ 3 ^)^ However, it is widely debated whether chrysotile asbestos can cause MM in humans, and this is mentioned in our article. ^(^ 1 ^)^ This controversy in the literature guides public policies in many countries, including Brazil. There is also controversy as to whether controlled exposure to low concentrations of chrysotile asbestos over a long period of time can result in detectable changes in humans.^(^ 4 ^)^ The issue is further complicated by the fact that chrysotile asbestos can be contaminated with crocidolite asbestos.^(^ 5 ^)^ Chrysotile asbestos accounts for 95% of all asbestos used worldwide, MM cases being therefore associated with this type of asbestos; however, it has been shown that there is no epidemiological evidence for a causal association between chrysotile asbestos and MM.^(^ 6 ^,^ 7 ^)^

There is no consensus in the literature regarding the effects of chrysotile asbestos exposure at low doses over a long period of time or at high doses over a short period of time on the development of MM.^(^ 4 ^-^ 8 ^)^ However, according to Dr. Algranti, there is no doubt about the carcinogenic effects of chrysotile asbestos in humans, and we respect his opinion. Nevertheless, in our opinion, there is no consensus! 

After a thorough study of the literature, our opinion is that there is no consensus regarding this issue. If there is indeed a consensus, why is it that chrysotile asbestos is still marketed in and exported by several countries, including Brazil? This is not true for amphibole asbestos; because there is a consensus on its role in the development of MM, it has been banned worldwide. Although the IARC classifies chrysotile asbestos as a carcinogen,^(^ 3 ^)^ several other groups and authorities worldwide have not been convinced by the currently available evidence; this further fuels the ongoing controversy. In addition to being scientifically controversial, the issue is controversial from an economic standpoint, given the large sums of money involved in worldwide asbestos markets. However, there is a consensus in the literature regarding the fact that the potency of chrysotile asbestos is lower than that of other forms of asbestos.^(^ 2 ^-^ 4 ^,^ 7 ^-^ 9 ^)^

We believe that our statement regarding chrysotile asbestos is correct; there is controversy, rather than consensus, regarding the role of chrysotile asbestos in humans. This is due to many factors, some of which have been mentioned above, whereas several others have been described elsewhere.^(^ 4 ^,^ 7 ^,^ 10 ^)^ Despite the controversy generated by several confounding factors in the aforementioned studies, the dangers of chrysotile asbestos cannot be ignored, which is why chrysotile asbestos and other types of asbestos are classified as human carcinogens. ^(^ 3 ^)^ This is clearly stated in our article.^(^ 1 ^)^ Finally, because our study focused on the molecular bases of MM, we decided not to delve into the controversy regarding the role of chrysotile asbestos in MM. However, the controversial nature of the issue is manifested in Dr. Algranti's letter and in our reply. 

We would like to thank Dr. Algranti again for his interest in our paper; for his critique; and for his bringing to light issues that are extremely important to the discussion of mesothelioma in Brazil. 

Yours sincerely,

Leonardo de Assis

Mauro César Isoldi
