# Supplementary material for: Authors' reply
Source: J Bras Pneumol. 2014 Sep-Oct;40(5):586–7. doi: 10.1590/S1806-37132014000500018 (PMC4263343; doi:10.1590/S1806-37132014000500018)
Supplement: Translation

# Translation: Resposta dos autores

**Authors:** Leonardo de Assis, Mauro César Isoldi

**Affiliations:** Instituto de Biociências, Universidade de São Paulo, São Paulo (SP) Brasil; Universidade Federal de Ouro Preto, Ouro Preto (MG) Brasil

Prezado Dr. Eduardo Algranti,

Agradecemos o interesse na leitura de nosso trabalho e também as críticas realizadas, as quais são de grande valia não somente para esse trabalho em particular, mas também para uma discussão mais ampla do mesotelioma maligno (MM) em nosso país. 

Iniciaremos nossa resposta relativa ao vírus símio 40 (SV40). No resumo,^(^ 1 ^)^ encontra-se a seguinte frase: ''O desenvolvimento do MM é fortemente correlacionado com a exposição ao amianto e erionita, assim como ao vírus símio 40''. De fato, tal afirmação na presente forma é errônea e concordamos com a crítica do Dr. Algranti.

Antes de o artigo ser editado pelo *Jornal Brasileiro de Pneumologia*, a seguinte frase se encontrava: "O desenvolvimento desse câncer é fortemente correlacionado com a exposição ao amianto e a outros fatores, como a erionita e o vírus SV40". Na frase original (antes da edição), a palavra "fortemente'' está relacionada com a exposição ao amianto e não relacionada ao amianto, erionita e SV40; contudo, reconhecemos que a mesma frase é dúbia e pode gerar dúvidas, dependendo da interpretação. Na frase original, a colocação de uma vírgula separando a palavra "amianto'' da palavra "erionita'' deixaria mais claro que a palavra ''fortemente'' está relacionada somente com a palavra "amianto''; contudo, ainda assim, a frase seria dúbia e passível de dupla interpretação.

Vários estudos têm associado fortemente o amianto ao desenvolvimento do MM. Agora, associar o SV40 fortemente com o MM é de fato errado e concordamos com as críticas do Dr. Algranti. Adicionalmente, em um trabalho recente de nosso grupo,^(^ 2 ^)^ consta a seguinte frase relativa à participação do SV40 no MM:

Taken altogether, it is still not clear the direct carcinogenic effects of SV40 in MM in humans; however, it is widely accepted the role of SV40 as a co-carcinogenic player in association with asbestos in the development of MM. 

Diante disso, reconhecemos que a frase inicialmente submetida era dúbia, sendo que durante o processo de edição, a mesma ficou mais clara, porém no sentido errôneo. De fato, colocar no mesmo nível o amianto e a erionita com o SV40 é errôneo e deve ser corrigido. Portanto, pediremos ao Jornal Brasileiro de Pneumologia a correção da frase que se encontra no resumo para a seguinte: "O desenvolvimento desse câncer é correlacionado com a exposição ao amianto e a outros fatores, como a erionita e o vírus SV40". 

A seguir, discutiremos a crítica relativa à participação do mineral crisotila no desenvolvimento do MM. Concordamos com a afirmação do Dr. Algranti relativa ao potencial inferior do amianto crisotila frente ao amianto anfibólio. De fato, no mesmo artigo,^(^ 1 ^)^ dizemos que todos os tipos de amianto são classificados como carcinogênicos em humanos pela IARC.^(^ 3 ^)^ Entretanto, é amplamente discutida a questão se o amianto crisotila é capaz de gerar MM em humanos, sendo que tal fato está descrito no artigo.^(^ 1 ^)^ Além disso, devido a essa controvérsia na literatura, muitos países utilizam isso como um fato norteador de suas políticas públicas, como é o caso do Brasil. Há controvérsia também se a exposição controlada ao amianto crisotila em baixas concentrações por um longo intervalo de tempo levaria a mudanças detectáveis em humanos.^(^ 4 ^)^ Além disso, sabe-se que minerais do tipo crisotila podem possuir contaminações de amianto crocidolita, trazendo mais um fator de confusão à questão.^(^ 5 ^)^ Outros argumentos conflitantes são os seguintes: 95% do amianto utilizado mundialmente é do tipo crisotila; logo, devido a sua presença quase absoluta no mundo, os casos de MM estão relacionados a esse tipo de amianto, ao passo que outros trabalhos demonstram que não há evidência epidemiológica que discrimine que, de fato, o amianto crisotila é o causador de MM.^(^ 6 ^,^ 7 ^)^

Levando todos esses fatores em consideração, não há consenso na literatura no que tange ao efeito do amianto crisotila, seja em pequenas doses durante um longo intervalo tempo, seja em elevadas doses em um curto intervalo de tempo no desenvolvimento do MM.^(^ 4 ^-^ 8 ^)^ Tal controvérsia não é compartilhada pelo Dr. Algranti, que acredita que não há dúvidas relativas ao efeito carcinogênico do amianto crisotila em humanos, sendo essa visão respeitada por nós autores; contudo, a nossa leitura da literatura é essa - não há consenso!

Dessa forma, nós, autores, através do estudo profundo da literatura, acreditamos que não há um consenso referente a esse tópico. Por fim, se de fato houvesse tal consenso, o qual é compartilhado pelo Dr. Algranti, então por que vários países do mundo, incluindo o Brasil, ainda comercializam e exportam o amianto crisotila? Essa prerrogativa não é verdade para o amianto do tipo anfibólio, para o qual há um consenso no seu papel no desenvolvimento do MM, fato que resultou no banimento desse tipo de amianto em praticamente todo o mundo. Mesmo em relação ao amianto crisotila, que é considerado como carcinogênico pela IARC,^(^ 3 ^)^ ainda há diversos outros grupos e autoridades de países que não foram convencidos pelas evidências demonstradas até o presente, o que alimenta ainda mais essa atual controvérsia. Além da questão científica, deve ser ressaltado que tal controvérsia é também sustentada pelo fator econômico, uma vez que o mercado de amianto mundial movimenta grandes quantias de dinheiro. Em contrapartida a tudo isso, há um consenso na literatura relativo à menor potência do amianto crisotila frente às demais formas de amianto.^(^ 2 ^-^ 4 ^,^ 7 ^-^ 9 ^)^

Acreditamos que nossa afirmação relativa ao amianto crisotila é correta, uma vez que não há consenso, bem como há grandes controvérsias nas informações relativas ao papel do amianto crisotila em humanos devido a inúmeros fatores, alguns mencionados acima e diversos outros que se encontram em outros artigos.^(^ 4 ^,^ 7 ^,^ 10 ^)^ Mesmo com tamanha controvérsia devido a vários fatores de confusão presentes naqueles estudos, não se deve ignorar o perigo do amianto crisotila, que levou à classificação do amianto crisotila e demais tipos de amianto em carcinogênicos em humanos.^(^ 3 ^)^ Tais fatos estão descritos de forma clara em nosso artigo.^(^ 1 ^)^ Por fim, como o foco de nosso artigo era as bases moleculares do MM, decidimos somente comentar tal controvérsia, a fim de informar os leitores da existência da mesma; contudo, sem entrar em detalhes, devido ao teor controverso do tópico, o qual se materializou na carta do Dr. Algranti e em nossa reposta aqui descrita. 

Agradecemos novamente ao Dr. Eduardo Algranti o interesse em nosso trabalho, pelas criticas e por trazer à luz questões de grande importância para o debate do mesotelioma em nosso país.

Cordialmente, 

Leonardo de Assis

Mauro César Isoldi
